# Recent Advances in Analytical Approaches for Glycan and Glycopeptide Quantitation

**DOI:** 10.1074/mcp.R120.002095

**Published:** 2021-02-20

**Authors:** Daniel G. Delafield, Lingjun Li

**Affiliations:** 1 Department of Chemistry, University of Wisconsin-Madison, Madison, Wisconsin, USA; 2 School of Pharmacy, University of Wisconsin-Madison, Madison, Wisconsin, USA

**Keywords:** mass spectrometry, posttranslational modification, glycosylation, glycopeptide, glycan, quantitation, metabolic labeling, isotopic labeling, isobaric labeling, chemical labeling, CHO, Chinese Hamster Ovary, CID, Collisional-Induced Dissociation, DDA, Data-Dependent Acquisition, DIA, Data-Independent Acquisition, ESI, Electrospray Ionization, ETciD, Electron Transfer/Collisional-Induced Dissociation, ETD, Electron Transfer Dissociation, EThcD, Electron Transfer/Higher-Energy Dissociation, FDR, False Discovery Rate, HCD, Higher-energy Collisional Dissociation, iTRAQ, Isotopic Tags for Relative and Absolute Quantitation, MALDI, Matrix-Assisted Laser Desorption/Ionization, MRM, Multiple Reaction Monitoring, PGC, Porous Graphitic Carbon, PRM, Parallel Reaction Monitoring, SCE, Stepped Collision Energy, SRM, Selected Reaction Monitoring, SILAC, Stable Isotopic Labeling of Amino Acids in Cell Culture

## Abstract

Growing implications of glycosylation in physiological occurrences and human disease have prompted intensive focus on revealing glycomic perturbations through absolute and relative quantification. Empowered by seminal methodologies and increasing capacity for detection, identification, and characterization, the past decade has provided a significant increase in the number of suitable strategies for glycan and glycopeptide quantification. Mass-spectrometry-based strategies for glycomic quantitation have grown to include metabolic incorporation of stable isotopes, deposition of mass difference and mass defect isotopic labels, and isobaric chemical labeling, providing researchers with ample tools for accurate and robust quantitation. Beyond this, workflows have been designed to harness instrument capability for label-free quantification, and numerous software packages have been developed to facilitate reliable spectrum scoring. In this review, we present and highlight the most recent advances in chemical labeling and associated techniques for glycan and glycopeptide quantification.

## Highlights

• Novel glycomic applications of label-free, metabolic, isotopic, and isobaric labeling quantitation
• Informatic tools for investigative quantitative glycomics
• Critical considerations for entry or expansion of quantitative glycomics
• Introduction of synthetically facile, cost-effective labeling technology

## Graphical Abstract

**Figure:**
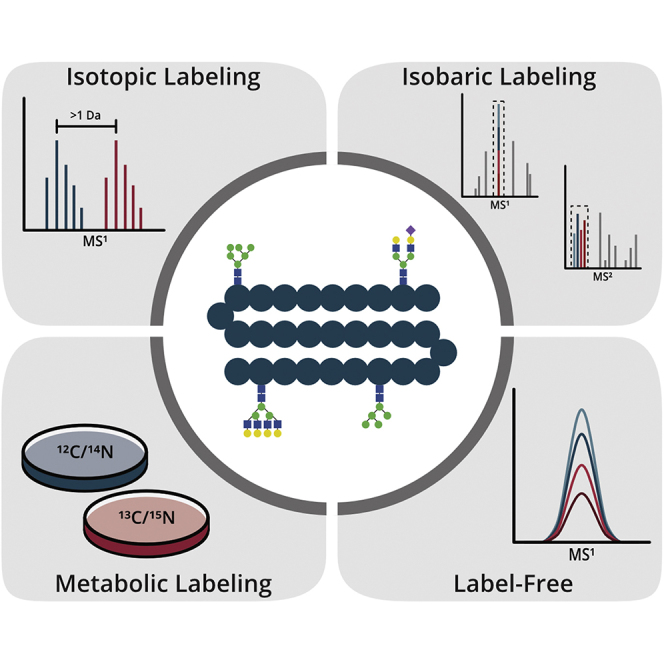

## In Brief

Recent years have seen an explosion in novel strategies for quantitative glycomics and glycoproteomics. Whether through metabolic incorporation of stable isotopes, deposition of custom isotopic labels, or high-throughput isobaric chemical tags, these numerous novel strategies provide ease of access to glycomic and glycoproteomic investigation. This review highlights the recent innovations in labeling methods, label-free strategies, acquisition modes, and bioinformatic tools for glycan and glycopeptide quantitation, while providing critical evaluations and technical considerations to enable effective analysis.

Continuous developments of analytical strategies enable advancements that illuminate the roles in which posttranslational modifications (PTMs) act to influence organism maturation, physiological processing, and immune response. While all members of this class of protein decorators are recognized for their alteration of protein function and contribution to proteomic diversity (1), no PTM is considered as complex or highly dynamic as that of glycosylation (2). The downstream products of enzymatic construction and deposition of carbohydrate moieties—glycans—onto a nascent backbone (2), glycoproteins present significant challenges in analysis due to their high degree of structural and compositional complexity (2), ionization inefficiency (3), low abundance (4), and the unique phenomena of macro- and microheterogeneity (2). Mass spectrometry (MS)-based glycomics has benefited greatly from advances in sample preparation protocols (5), enrichment strategies (6, 7, 8), and instrumental capabilities (fragmentation, data-dependent and data-independent acquisition (DIA), parallel reaction monitoring (PRM) etc.) (9, 10), which now provide broad access to the glycoproteome.

**Fig. 1:**
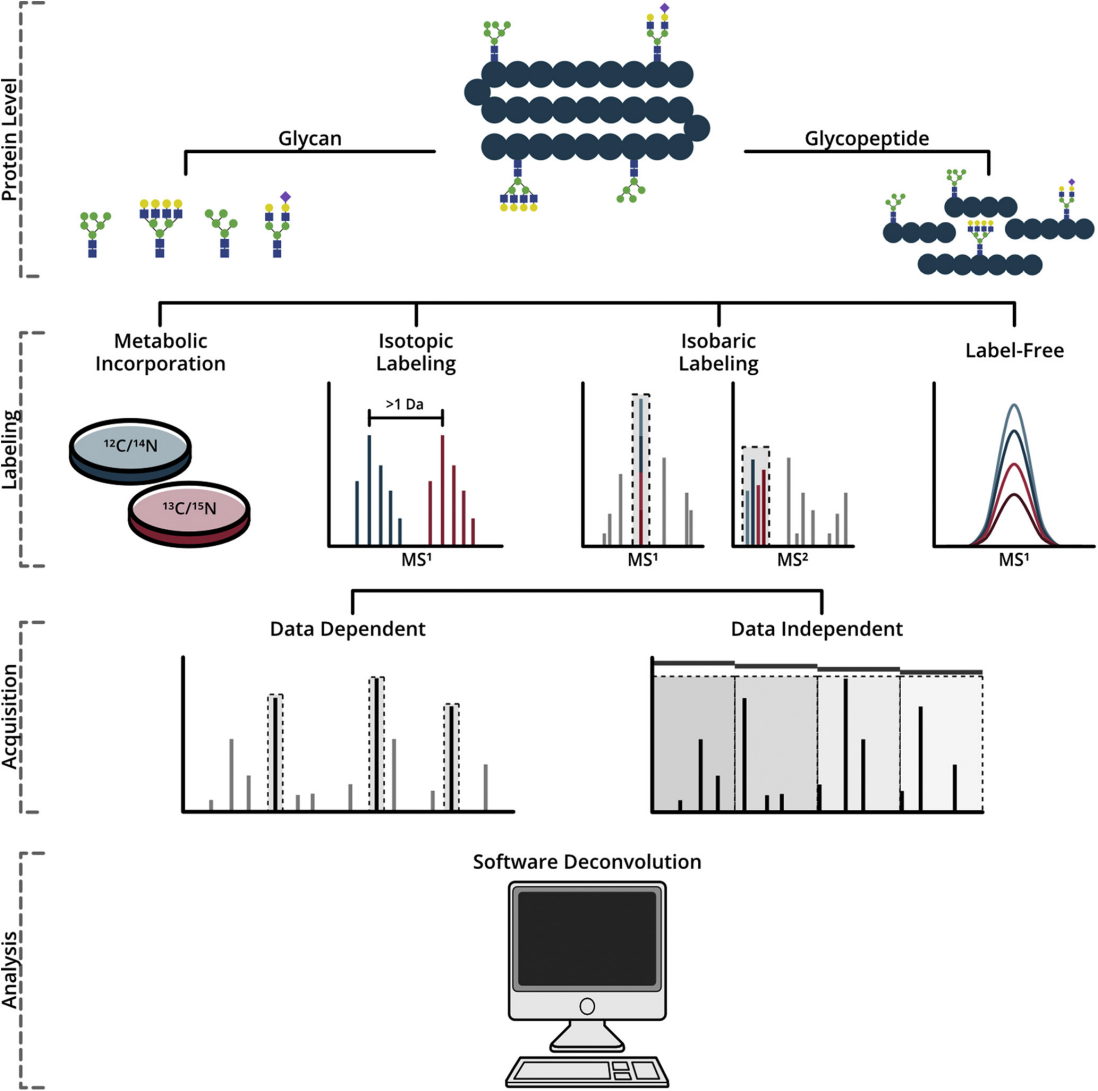
**Graphical representation of quantitative glycomics and glycoproteomic analyses.** Glycomic evaluations, as discussed here, may take place at either the glycan or glycopeptide level and pursued through incorporation of stable isotopes, deposition of isotopic labels for MS^1^ level quantification, isobaric labeling for MS^2^ level quantitation, or label-free comparison. Both data-dependent and data-independent acquisition are effectively employed for glycome or glycopeptide detection with numerous software tools available to perform identification and quantitative analysis.

As a result of these advances, targeted glycomic research continuously expands the implication of glycosylation in physiological processes such as cell signaling (11, 12, 13, 14), host–pathogen interaction (15, 16, 17, 18), and immune response (11, 19, 20, 21), with significant revelations provided in connection to human disease. Recent evaluations demonstrating the importance of glycosylation in neurodegenerative diseases (22, 23), diabetes (24, 25), and cancer (26, 27, 28) promote further interest in glycomic investigation to reveal potential biomarkers and unambiguous symptomatic protein profiles. As focus shifts from glycomic discovery and characterization to that of glycan expression levels and minute perturbations in site occupancy, the need for robust and efficient glycan and glycopeptide quantitative strategies steadily grows. In response to this demand, the last decade has seen a surge in reports detailing novel chemical-labeling-based and label-free strategies built on both data-dependent and data-independent acquisition for quantitative glycomics (Fig. 1). The previous review by Mechref *et al.* (29) provides a detailed discussion of the seminal reports paving the way for recent innovations, which may be explored in supplement to the strategies outlined herein. Discussed below are the most recent advances in metabolic incorporation, isotopic and isobaric chemical labeling, label-free approaches, and software for quantitative glycan and glycopeptide analysis.

## Glycan Quantitation

As glycoconjugate function is shown to be impacted by glycan structure and composition, enzymatic or chemical release of glycans provides direct access to profiling altered glycan expression while enabling structural and compositional characterization. Considering the ever-present challenges in glycan analysis such as ionization inefficiency, highly hydrophilic character, glycosidic bond lability, and presence of negative charge, effective glycan quantitation may be achieved through strategies that offer reprieve from these ailments while providing facile labeling and reduction in spectral complexity.

### Isotopic Labeling

Glycan quantification at the MS^1^ level is an attractive prospect due to broad access to higher resolution instrumentation and the reduced considerations of selectivity bias in data-dependent acquisition (DDA) experiments. Relative quantitation in this manner is often achieved through labeling of glycans in “heavy” and “light” channels to produce a consistent mass difference (*i.e.*, >1 Da). In order to avoid retention time differences between constituents of each channel and increase quantitative accuracy, heavy and light labels are engineered through the incorporation of stable isotopes, such as ^12^C and ^13^C. 2-aminobenzoic acid (2-AA) is a classic glycan label, often employed for its fluorescent properties in UV-based experiments (30) and was adapted for isomer-specific quantitative glycomic evaluations (31). The well-characterized labeling strategy, commercial availability of isotopologues, and complete separation of isotopic envelopes—necessary to avoid peak overlap and inaccurate quantitation—make this strategy well-suited for facile quantitation. The importance of envelope separation was reinforced in the preliminary report of glycan reductive isotopic labeling (GRIL) (32), which employed aniline isotopologues to stabilize sialic acid linkages, eliminate negative charge, and distinguish isotopic envelopes. GRIL was later employed for glycan analysis through porous graphitic carbon (PGC) LC-MS, which enabled liquid-phase resolution of biantennary sialylated glycans (33, 34). CID fragmentation was shown to provide antennae-specific fragmentation, further indicating the ability to quantify differential expression of isomeric glycans. Additionally, Walker *et al.* established a method labeling glycan with isotopic hydrazide tags (35), INLIGHT (36), which echoes the importance of envelope separation to eliminate inaccurate isotope correction or quantitation. This method was validated against glycan standards and those extracted from human plasma, demonstrating quantitative accuracy across four orders of magnitude.

As an alternative to carbon isotopes, glycans may be labeled with heavy oxygen (^18^O) when enzymatic release is performed in the presence of heavy water. First reported by Tao and Orlando (37), the mechanism of glycan release with PNGase F results in a terminal amine group at the glycan reducing end, which is then replaced with a hydroxyl group after spontaneous hydrolysis. When released in heavy water, glycans will express a 2 Da mass shift over unlabeled counterparts. This method has been further applied (38) and is advantageous in that it requires no synthesis or treatment with commercial isotopologues and that labeling efficiency is at or near 100%, depending on the purity of heavy water available. However, considering sample complexity and the unavoidable overlap of isotopic envelopes when labeled/unlabeled pairs are separated by only 2 Da, Cao *et al.* (39) developed a strategy for glycan reducing end dual isotopic labeling (GREDIL), which provided an additional 1 Da mass shift through NaBH_4_/NaBD_4_ reduction of glycans.

Beyond heavy carbon and oxygen, the incorporation of deuterium has been widely reported in quantitative glycomics experiments. As glycan permethylation (40) is routinely employed to reduce the high hydrophilicity of glycans and increase ionization efficiency prior to LC-MS analyses, early reports demonstrate simple workflow adaptation using iodomethane isotopologues to produce three labeling channels through light, medium, and heavy methyl labels (*i.e.*, CH_3_, CD_2_H, CD_3_) (41). The same research group later expanded this workflow into an eight-plex labeling strategy that included additional heavy carbon isotopes (42). Early reports of deuterium-based isotopic tags were provided by Bowman and Zaia, who first assessed multiple novel compounds for tetraplex labeling (43) and later applied them for glycan and glycosaminoglycan quantitation (44). Numerous other deuterium-based isotopic labeling strategies have been employed: derivatization with phenyl-methyl-pyrazole (PMP) has been employed to produce a one-pot dual-channel labeling strategy for matrix-assisted laser desorption/ionization (MALDI)-based quantitation of O-glycans (45, 46), which was also adapted for in-gel labeling without significant sample loss (47); stabilization and quantitation of sialic-acid-containing glycans was promoted through a solid-phase p-toluidine labeling strategy (48); duplex stable isotope labeling (DuSIL) was developed to discriminate neutral and sialylated glycans without the need for synthesis (49, 50, 51); isomer-specific quantitation of sialic-acid-containing glycans was achieved through Glycoqueing, which enabled sialoglycan stabilization, isomer-specific elution order, and boosted MS signal (52); and quantitation by mutant enzyme reaction stable isotope labeling (QMERSIL) facilitated glycan release and labeling in a single step (53). Other methods for MS^1^ level quantitation are reported by Yang *et al.*, (54) who employed a metal chelating agent (p-NH_2_-Bn-DOTA) and rare earth metals to provide a 10 Da mass shift and near 100% labeling efficiency, and the quantification of N-glycan types presented by Li *et al.* (55) that couples endoglycosidase digestion with channel labeling to provide an enrichment-friendly three-plex labeling strategy composition.

Due to the significant sample handling necessary for glycan purification, derivatization, labeling, and cleanup prior to electrospray ionization (ESI)-based MS experiments, Chen *et al.* conceived a strategy that leverages the salt-tolerant, facile nature of MALDI-based glycan analysis while eliminating the ion suppression that stems from sample complexity. Combining glycans after labeling with light/heavy HDEAT (2-hydrazino-4,6-bis-(diethylamino)-s-triazine)—which provides a 20 Da mass shift between species, HILIC separation was employed to deliver a liquid trace onto a MALDI plate. After matrix application, the liquid trace could be analyzed directly to identify N-glycans. The spatial distribution of glycans on the MALDI plate could be reconstructed into a base peak chromatogram to provide retention time of glycan species. This method reports significantly improved performance for glycan quantitation with higher sensitivity, reproducibility, and accuracy compared with MALDI alone and may be further expanded to multiplexed experiments (56).

**Fig. 2:**
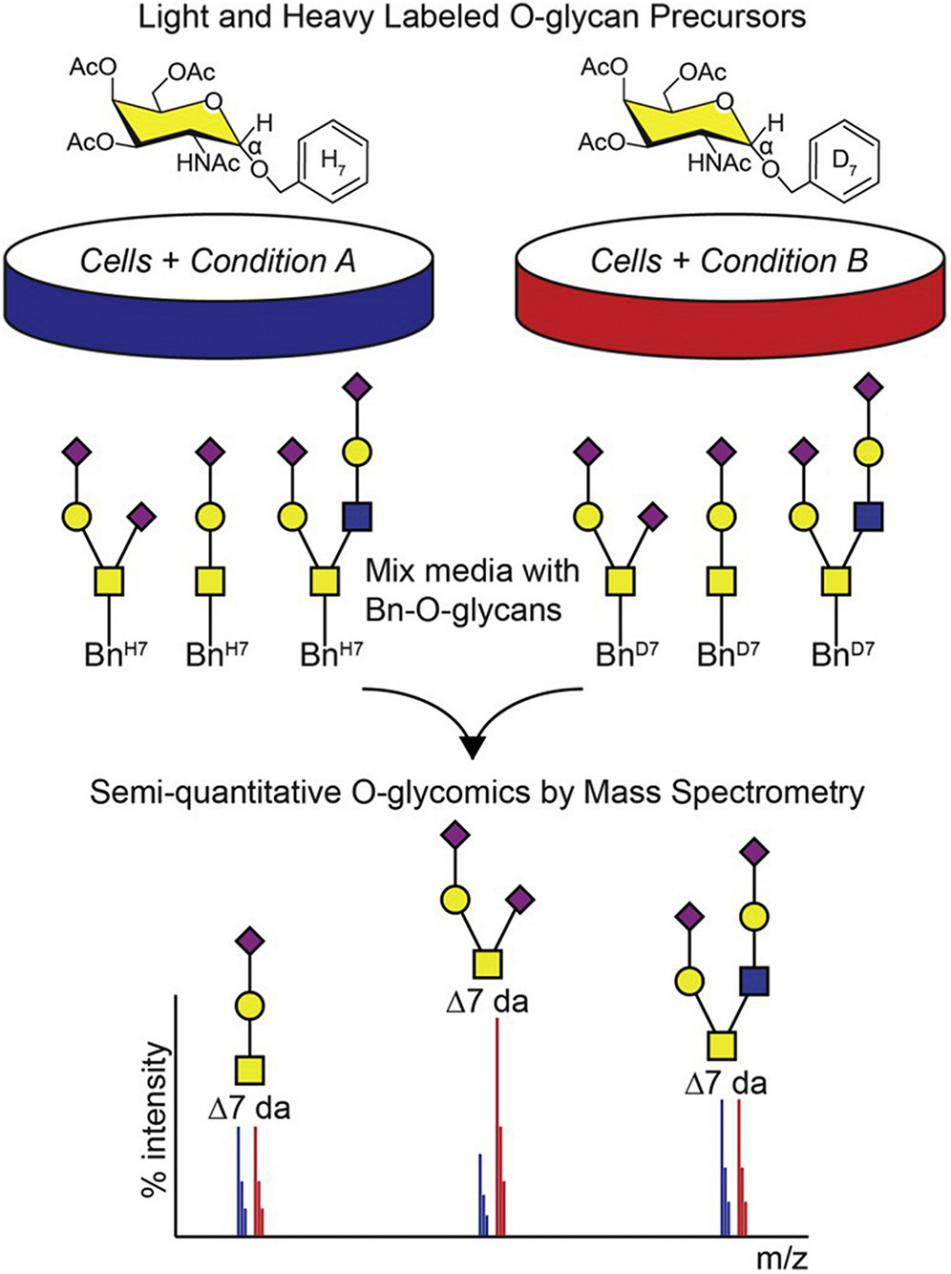
**Overview of isotopic labeling with cellular O-glycome reporter/amplification (ICORA).** Cells undergoing condition A are incubated with Ac3GalNAc-BnH7 while cells undergoing condition B are incubated with Ac3GalNAc-BnD7. Ac3GalNAc-Bn crosses the plasma membrane, is de-esterified in the cytosol, taken up into the Golgi apparatus, and modified by endogenous glycosyltransferases to produce light H7 or heavy D7 labeled Bn-O-glycans before being secreted into the media. Media from the two conditions is mixed together and heavy and light Bn-O-glycans are purified, permethylated, and analyzed by mass spectrometry. A 7 Da mass shift distinguishes the light and heavy O-glycans, enabling quantification of shifts in relative abundance and comparison of O-glycans in condition A *versus* condition B. Reprinted from Kudelka *et al.* (58) with permission from the author.

Of particular note are strategies that reduce sample handling and associated loss by employing cellular machinery to facilitate glycan labeling, combining features of both metabolic and isotopic labeling. A pioneering study of this kind was provided by Kudelka *et al.*, (57) who introduced cellular O-glycome reporter/amplification, CORA. This methodology involves the supplementation of cell culture media with paracetylated benzyl-α-N-Acetylgalactosamine (GalNAc-Bn), which is extended into a mature glycans by endogenous glycosyltransferases. Because the reducing end of the glycan is blocked by the benzene group, these glycans are not acted on by oligotransferases, rather being excreted from the cell and escaping degradation. The benzene group also facilitates simple purification using reversed-phase cartridges for efficient MS analysis of the O-glycome constituents. This method was further developed to enable relative quantitation by employing light/heavy GalNAc-Bn in the method dubbed ICORA, isotopic labeling with cellular O-glycome reporter/amplification (58) (Fig. 2). Highlights of this method include complete discrimination of isotopic envelopes through a 7 Da mass shift, high levels of persistence found in Bn-protected glycans, and the ability to evaluate O-glycome perturbations in response to altered growth conditions. Though this method does not mitigate any of the challenges in glycan analyses (*e.g.*, MS/MS of low abundance species, structural assignment, or accuracy of MALDI *versus* ESI) and is not broadly useful beyond MS due to the weak absorbance of the benzene ring, this method does provide a rigorous example of how “classic” metabolic incorporation of stable isotopes and azide sugars may be employed for glycan amplification and quantitation—an idea expanded much further in quantitative glycopeptide experiments (vide infra).

### Metabolic Incorporation

Though isotopic labeling is successfully employed for MS^1^ level comparison of glycans, the questions of labeling efficiency, as well as reagent synthesis, cost, and availability remain. As an alternative, several researchers have turned to the classic strategy stable isotopic labeling of amino acids in cell culture (SILAC), which significantly reduces concerns over labeling efficiency while retaining the ability to perform relative quantitation and offering a means to discern glycome lifetime and stability.

IDAWG, isotopic detection of amino sugars with glutamine (59), is one of a few seminal reports on the feasibility and accuracy of metabolic incorporation for relative quantitation. Though discussed in depth in the previous review (29), briefly, heavy nitrogen was introduced to cell culture in the form of ^15^N-glutamine, which provided near-complete labeling of glycosylation sites and aminosugars across the observed proteome. This method demonstrates the reliability of metabolic incorporation for glycosite and glycan quantification, as well as how media treatment can be used to evaluate further synthesis or degradation of aminosugar-containing glycans in response to cellular behavior. This idea was further expanded by two groups who sought to comprehensively quantify the glycome and glycoproteome through combining metabolic incorporation and isotopic labeling. Yang *et al.* (60) accomplished characterization of bladder cancer cell lines (KK47, YTS1, J82, T24) against a normal bladder mucosa cell line (HCV29). This report employed SILAC labeling for proteomic quantification while combining lectin microarrays and sialylated glycan derivatization with heavy/light aniline to comprehensively quantify glycan expression levels. Further expansions of combinatorial methods are provided in the report of solid-phase extraction of N-linked glycans and glycosite-containing peptides (NGAG) by Sun *et al.* (61). This method employs sequential elutions after tryptic peptides have been complexed with aldehyde-functionalized resin beads. In the first pass, lysine side chains are protected through guanidination prior to derivatization of acidic species (sialic acid and aspartic acid) with aniline, which is followed by PNGase F treatment to release N-glycans. The released glycans were then labeled with iTRAQ, isotopic tags for relative and absolute quantitation, prior to LC-MS identification and quantitative analysis. In the second pass, the newly formed aspartic acid residues that result from glycan release are then cleaved by Asp-N treatment, eluted, and quantified after combining with heavy-labeled glycosite-containing peptides from SILAC treatment. Using the NGAG method to analyze OVCAR-3 Cells, 85 unique glycan compositions and 2044 glycosite-containing peptides were identified, offering complementary coverage to that of the previously reported SPEG methodology (62) of the same group. These methods present an efficient strategy for quantifying the glycome and glycoproteome through metabolic incorporation of stable isotopes, providing an avenue of expansion, which has since been greatly explored in quantitative glycopeptide experiments. However, given the lack of suitable stable isotopes that may be incorporated and the increasing spectral complexity when numerous isotopes are present, these mass-difference experiments are fundamentally limited by the number of channels that may be analyzed at any one time. As such, great benefit may be found in employing the strategy of mass-defect-based chemical labels.

### Mass Defect

**Fig. 3:**
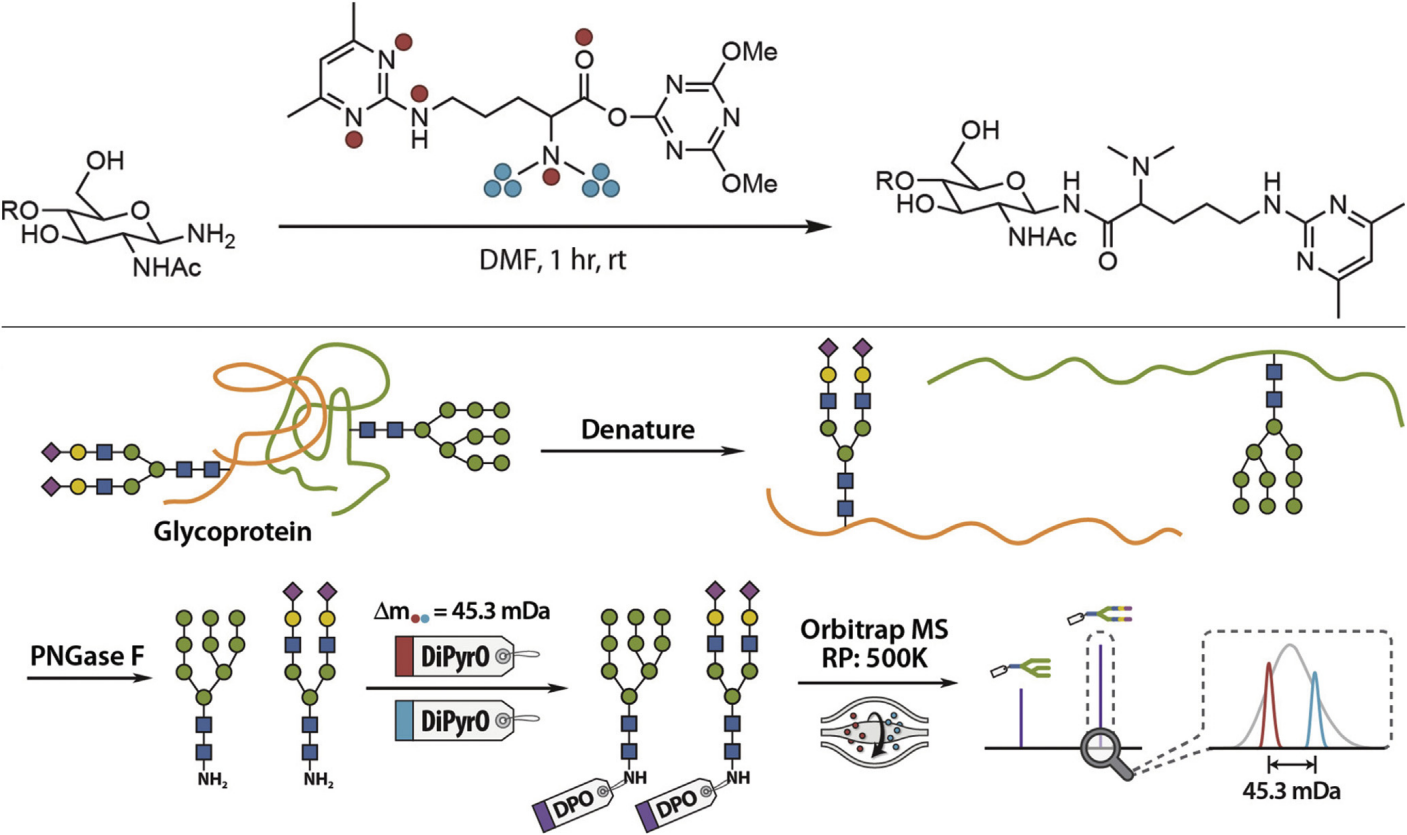
***Top*, DiPyrO labeling of glycosylamine; *Red dots* represent heavy isotopic atoms (**^**15**^**N**_**4**_^**18**^**O) in the light DiPyrO tag; *blue dots* represent heavy isotopic atoms (**^**2**^**H**_**6**_**) in the heavy DiPyrO tag.***Bottom*, workflow for the relative quantification of DiPyrO-labeled N-glycans illustrating the microenvironment. Adapted from Chen *et al.* (66) with permission.

While isotopic labeling and metabolic incorporation impart a mass shift of >1 Da—mass-difference, mass-defect-based strategies impart mDa mass shift. As such, MS^1^ mass spectra are significantly less complex than in mass difference experiments, redundant sampling is avoided because all labeled ions are selected for fragmentation in the same MS^2^ isolation window, and quantification at the MS^1^ level is retained, reducing the concerns over precursor coisolation. Early implementations of such strategies using CH_3_I and CH_2_DI have been reported (63, 64), but few reports exist over recent years. One example provided by Chen *et al.* was the successful application of mass defect dimethyl pyrimidinyl ornithine (DiPyrO) (65), an amine reactive tag, for quantitative glycomics (66) (Fig. 3). This study successfully quantified glycan expression differences between B-cells of healthy and acute lymphoblastic leukemia and demonstrated dynamic linearity across two orders of magnitude. This study provides two notable observations: i) increasing instrumental resolution will facilitate immediate expansion of DiPyrO beyond three demonstrated labeling channels and ii) employing amine reactive tags for glycan quantitation is a promising path that can be widely explored. This latter notion was explored by Feng *et al.* (67) in the development of mass-defect isobaric multiplex labeling reagents for carbonyl-containing compound (mdSUGAR) tags. This three-channel approach was built upon the simple three-step synthesis of the original SUGAR tags (68) (see below), providing a 23.8 mDa mass shift between channels and labeling at both the reducing end and on sialic acid residues for stabilization. Beyond the significant reproducibility demonstrated when analyzing standard and complex samples, the MS^2^ fragmentation spectra revealed complete *y* glycan fragment series with the mdSUGAR tag attached with additional tagged *b* ions found in sialylated glycans. This improved fragmentation series compared with unlabeled species allows for greater confidence during glycan annotation and structural assignment. These approaches represent a facile strategy for glycan labeling, with excellent accuracy and dynamic range that can be employed in scenarios where instrument resolving power is limited. Further expansion of these tags may prove useful in highly multiplexed experiments that seek to exploit rapidly evolving capabilities of novel instrumentation.

### Isobaric Labeling

In order to avoid explosions in spectral complexity and the need for slower, higher-resolution MS^1^ scans, numerous reports have explored the utility of glycan quantitation at the MS^2^ level. By employing isobaric labels—each of which has an identical overall mass but a reporter ion region of unique mass—collision-based dissociation allows for relative quantitation to proceed through the comparison of reporter ion abundance.

**Fig. 4:**
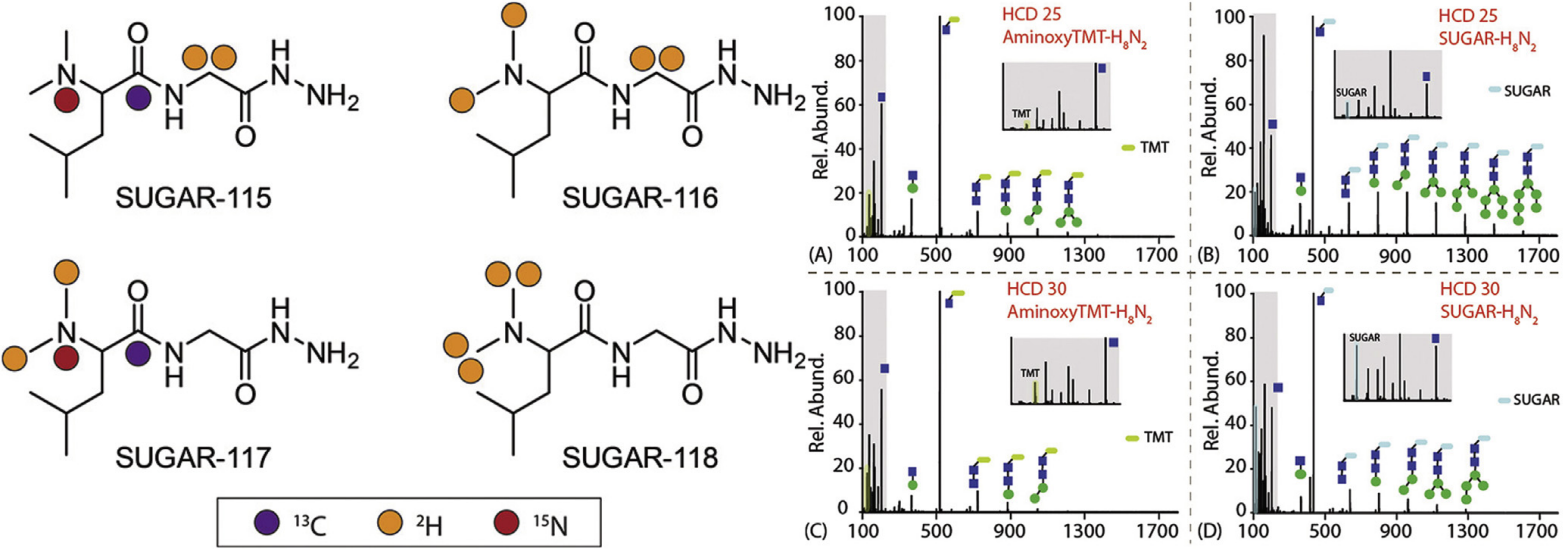
***Left*, structure and isotope configurations of four-plex SUGAR tags.***Purple dot*: ^13^C, *orange dot*: ^2^H, *red dot*: ^15^N. *Right*, ESI-MS/MS fragmentation comparison of aminoxyTMT-labeled and SUGAR-labeled N-glycans. AminoxyTMT-labeled H_8_N_2_ ([aminoxyTMT − H_8_N_2_ + 2H]^2+^) at NCE 25 (*A*) and 30 (*C*), SUGAR-labeled H_8_N_2_ ([SUGAR − H_8_N_2_ + 2H]^2+^) at NCE 25 (*B*) and 30 (*D*). Adapted from Feng *et al.* (68) with permission.

At the time of last review, isobaric labeling strategies for glycan quantitation were only just emerging. iART, isobaric aldehyde reactive tags, was an early report of MS^2^ based quantitation, employing a simple synthesis strategy to create two labeling channels. This method demonstrated significant improvements in glycan sensitivity postderivatization as well as reliable quantitation when applied to quantifying the gp120 subunit of the HIV envelope (69). The same researchers later expanded this underlying strategy in developing a four-plex labeling strategy by developing quaternary amine containing isobaric tag for glycans, QUANTITY (70). This method was originally validated using N-glycans released from human serum and CHO cell lines, which revealed relative quantitation of 90 and 159 N-glycans, respectively. Later, QUANTITY was employed for simultaneous quantitation of N- and O-glycans through sequential release and labeling techniques (71). Concurrent with these studies, numerous strategies were established for glycan quantitation using commercial tandem mass tags (TMT). Though glycoTMT, a carbonyl reactive tag for N-glycan quantitation, was reported early (72), broad applicability was demonstrated through the use of the amine reactive tags, aminoxyTMT (73, 74, 75, 76, 77). Notably, Zhong *et al.* (74) demonstrated baseline resolution of TMT-labeled high-mannose glycans through capillary electrophoresis, while CE-TWIM-MS (capillary electrophoresis–traveling wave ion mobility–mass spectrometry) was able to distinguish isomers of sialylated O-glycans in human milk. Chen *et al.* (76) later established the improved quantitative accuracy of N-glycans using MultiNotch MS^3^ triggered by the presence of Y_1_ glycan ions. These recent reports indicate the utility of isobaric labeling for deep glycomic quantitation; however, the inefficiency of multistep syntheses presented by iART and QUANTITY, as well as the high cost of commercial TMT labels, often places these workflows out of reach. In remedy to this, Feng *et al.* (68) developed Isobaric Multiplex Labeling Reagents for Carbonyl-Containing Compound (SUGAR) tags (Fig. 4). This report details a simple, three-step synthesis of SUGAR isotopologues with ∼70% overall yield, and two-step labeling for near 100% labeling efficiency of all N-glycans tested. As well, the low cost of the reagents employed makes this an attractive strategy that may be readily implemented in numerous research settings. Finally, in addition to the efficiency and quantitative accuracy, SUGAR tags demonstrated significantly improved glycan fragmentation in CID/HCD-based experiments for more accurate structural and compositional assignment. Considering these numerous developments over recent years, isobaric labeling is seen as an effective strategy for glycan quantification, which is likely to be further expanded with improvements in instrument resolution and need for increased sample throughput.

### Fluorescence Labeling

Fluorescence and absorbance-based labeling strategies were methods of significant interest prior to the heavy development of MS-based technology and MS-suitable sample preparations. However, fluorescence labeling is still employed due to the relative ease of glycan derivatization, the reduced need for intensive sample cleanup, and the reduction of sample loss *via* reduced sample handling. A notable improvement in glycan labeling efficiency was reported by Lauber *et al.*, (78) where they demonstrated that commercial RapiFluor-MS can label glycans in under 5 min compared with the >1 h found strategies mentioned above. RapiFluor-MS also facilitated quantitative recovery of glycans during cleanup, facilitated sensitive fluorescence, and quantitative accuracy in ESI-MS experiments. In the effort to reduce the limitations surrounding single-channel measurements of fluorescence-based strategies, Rana *et al.* (79) developed a three-channel sensing system that employs unique fluorescent proteins to generate a multiplex output. Utilizing gold nanoparticles with a glycan recognizing functional ligand, this strategy proved useful in rapidly and quantitatively comparing human cell types according to their surface glycan profiles.

### Label-Free

Rapidly evolving instrumental capabilities present a unique path toward quantitative glycomics. An ideal approach to quantitative experiments is the incorporation of an internal standard, but this method is not widely employed due to the complexity of glycan synthesis and the lack of commercial isotopic glycan standards. iGlycoMab, an isotope-labeled monoclonal antibody, was recently developed through ^15^N metabolic incorporation. As heavy nitrogen will be incorporated into the aminosugars of the single glycosylation site on the Fc region, glycans released from this standard protein can serve as an internal isotopic standard in glycomics experiments. This strategy was successfully employed by Zhou and others, indicating the feasibility of isotopic glycans as internal standards (80). An alternative strategy using the incorporation of exogenous standards was also validated for glycan quantitation (81). As data suggests that molar responses for permethylated glycans are relatively uniform, investigators spiked in permethylated malto-series glycans at known concentrations for absolute quantification of N-glycans. These two previous reports are unique strategies for glycan quantification, but both state the need for a complete N-glycan standard series for more accurate, reliable, and broadly useful experiments. Given the unavailability of isotope-encoded glycan standards, a premium is placed on methods capable of accurate quantitation while reducing dependence on internal standards. To this end, numerous reports have validated significant increases in analytical sensitivity and quantitative accuracy when employing parallel and multiple reaction monitoring.

### MS Reaction Monitoring

With rapidly expanding access to instrumentation capable of deciphering highly complex mixtures, alongside the appreciation of reliable and reproducible instrument performance, a growing number of investigators have sought to exploit instrument capabilities for absolute and relative quantitation. Rapidly gaining favor in the area of glycan analysis are selected, parallel, and multiple reaction monitoring (SRM, PRM, and MRM). Though each has been successfully employed for glycomic quantitation, MRM analyses have gained favor in glycoproteomics (82) due to more precise quantitation (83), high analytical reproducibility, better signal-to-noise ratios, and increased dynamic range (84). In-depth description of reaction monitoring concepts and considerations may be read elsewhere (85, 86). In brief, MRM, which is often implemented on triple quadrupole (QQQ) instrumentation, involves scanning of glycans in the first quadrupole, CID fragmentation in the second, and scanning of transitions (*i.e.*, fragments of precursor masses) in the third quadrupole. User control over valid precursor and transition masses results in a highly selective and sensitive method for glycan identification. Noting that transition signal response is directly related to analyte concentration, iterative analyses of standard mixtures can be employed to develop calibration curves of transition abundance. After analysis of unknown sample mixtures, these curves are used to provide absolute abundance of targeted analytes. The targeted nature and considerable effort needed to establish effective MRM workflows limit their utility in high-throughput experiments, but these techniques are widely useful in glycan biomarker and protein characterization studies (85).

Of the numerous reports employing reaction monitoring, Lebrilla and colleagues have been instrumental in developing novel methods for MRM analysis of mono- and oligosaccharides. For example, Hong *et al.* (87) detailed the ability to perform label-free absolute quantitation of human milk oligosaccharides (HMOs) and leverage 2’-fucosylation concentration to profile samples from secretors and nonsecretors. Of note, this method established quantitative accuracy across five orders of magnitude and displayed femtomole sensitivity, rearticulating the benefits of targeted MRM analyses. Later, Xu *et al.* (88) expanded on this approach and demonstrated that differences in retention time between monosaccharide isomers can be leveraged to create dynamic multiple reaction monitoring methods—a concept discussed in detail in later sections. In addition to these fundamental reports, Xia *et al.* (89) provided an early entry through their analysis of N- and O-glycans for diagnosis of congenital disorders. Later Tao *et al.* (90) reported a penta-HILIC-SRM-MS for the separation and identification of 2,3/2,6 sialic-acid-containing N-glycan isomers, and Tsai *et al.* (91) established a protocol for N-glycan biomarker discovery in hepatocellular carcinoma (HCC). MRM has also been used in combination with glycan permethylation to quantify 88 N-glycans from only 5 nl of human blood (92). Finally, Orlando *et al.* (93) have pursued absolute N-glycan quantitation of biotherapeutic antibodies, and Mank *et al.* (94) expanded on the earlier reports of HMO analysis to provide structural selectivity. These reports are among those that signal increasing interest in label-free, instrument-dependent methodologies for glycomic quantitation. Though the benefits and drawbacks of these strategies must be carefully weighed against those mentioned for chemical labeling, future innovations in the area of MS reaction monitoring and instrument efficiency could pave the way for a gradual shift toward confident and reliable label-free analyses.

### Critical Evaluations and Considerations

Numerous strategies have been developed for glycan quantitation, presenting unique benefits and drawbacks that must be considered prior to implementation. A guiding consideration should include relative sample complexity and need for throughput. In low-complexity experiments where throughput is not needed (*i.e.*, analyzing no more than two samples), isotopic labeling is an effective strategy that may be customized to fit individual needs. Isotopic labeling reveals greater benefits when employing tags that increase glycan hydrophobicity and ionization efficiency or impart positive permanent charge. As sample complexity increases, mass-defect-based isotopic labeling strategies may be implemented to offer reprieve from precursor coisolation and spectral complexity while also providing slightly higher throughput. In high-throughput investigative experiments, if samples are relatively simple and MS1 level quantification is possible, eight-plex glycan permethylation would be of use due to the significant increases in glycan hydrophobicity for LC separations and improved ionization efficiency. However, isobaric labeling is undoubtedly the method of choice in high-throughput, high-complexity experiments as quantitation is pursued in tandem with identification at the MS2 level. If seeking to perform analyses at this level, channel multiplexing, synthetic capacity, and cost will be the guiding factors. A brief summary of highlighted methods may be found in Table 1. No matter the application, the techniques presented above provide achievable avenues to those seeking to perform quantitative glycomic analyses.

**Table 1:** Comparisons of labeling strategies for glycan quantitation

Type	Method name	Pros	Cons
Metabolic incorporation/isotopic labeling	ICORA (58)	Improved reporting signal through increased O-glycan abundance, increased enrichment efficiency, optimal labeling efficiency	Only validated for O-glycans, time-restrictive, growth conditions must be carefully monitored
Isotopic labeling	Dimethyl labeling	Low-cost reagents, facile labeling, slight increase in glycan hydrophobicity	Limited throughput (low multiplexing capacity)
Isotopic labeling	Isotopic permethylation (42)	Significant improvements in glycan hydrophobicity and ionization efficiency, eight-channel multiplexing	Toxicity of iodomethane reagents
Isotopic labeling	Custom tags (*e.g.*, PMP-, Gerard’s reagent P-, aniline-based etc.)	Highly customizable, effective in bespoke tagging workflows, stabilization of sialic acid residues, fixing of permanent positive charges	Concerns over labeling efficiency, need for optimization and method design
Mass defect	DiPyrO (66)	Greatly reduced spectral complexity, elimination of redundant sampling, precursor coisolation does not affect quantification, amine reactive tag (may be applied to glycans, peptides, and proteins)	Low multiplexing capacity (3-channels), requires higher-resolution MS^1^ scans, current instrumentation outperforms multiplexing capacity
Mass defect	mdSUGAR (67)	Labeling at glycan reducing end and on sialic acids, improved glycan fragmentation compared with commercial tags	Carbonyl-reactive tags are not as flexible in peptide and protein quantification, offer three-channel multiplexing
Isobaric labeling	QUANTITY (70)	Improved fragmentation and reporter ion signal, high labeling efficiency. Quaternary amin imparts permanent positive charge	Requires multistep synthesis, offers four-channel multiplexing
Isobaric labeling	TMT	Commercial quality control, well-characterized protocols, eight-channel multiplexing, fits within Thermo “ecosystem”	Cost-preventative
Isobaric labeling	SUGAR (68)	Improved b/y glycan fragment series for identification, synthesized in three high-yield steps, near 100% labeling efficiency, higher reporter ion signal for quantitation	Offers four-plex multiplexing

## Glycopeptide Quantitation

Direct glycan analysis after enzymatic or chemical release enables understanding of the heterogeneity found within a given glycoproteome while providing the best opportunity for structural and compositional interrogation. In pursuit of comprehensive glycoprotein characterization, glycan analysis is limited by the elimination of protein localization as no glycan can be related to a modification site without intensive experimental control. To this end, analysis of intact glycopeptides retains site-specific information while enabling modest elucidation of the attached glycan. Though traditionally limited due to low abundance within proteolytic mixtures and poor ionization efficiency, glycopeptide analyses have benefited greatly from recent advances in sample preparation (95, 96, 97), enrichment strategies (98, 99, 100), and instrumental functionality (101, 102, 103). Enabled by broad access to the glycoproteome, revealing deviations at the glycan, modification site, and protein level are of immediate interest in the effort to provide a more comprehensive view that helps to elucidate the role of glycosylation in physiological processes and human disease. As the following reports exercise analysis of glycosylated peptides and deglycosylated peptides, clear distinction has been provided to avoid confusion. Discussion of “glycopeptides” refers strictly to glycosylated species, and all references involving release of glycans prior to analysis are noted as “deglycosylated peptides.”

### Metabolic Incorporation

As SILAC experiments involving the incorporation of heavy amino acids—traditionally heavy lysine and arginine—during protein translation, glycopeptide quantitation through metabolic incorporation is widely accessible. This approach was taken in early reports that detailed the utility of DIA of sequential isolation windows (SWATH-MS) for glycopeptide quantitation (104). DIA analyses will be discussed further in subsequent sections, but this initial report demonstrated the sensitivity and reproducibility gained during application. Further application of heavy amino acids was reported by Poljak *et al.*, (105) who used enzymatic cleavage and PRM of glycopeptides to quantitation the N-glycosylation machinery in yeast, though this method did not provide evaluation of glycan expression levels. While the applicability of incorporating isotopic labels is plainly seen across proteomics, significant contributions to glycopeptide analysis have come through the development of methods that combine efficient enrichment and complete labeling. Though the following methods enable quantitation through isotopic labels, they are presented here for their unique implementation of metabolic azide sugar incorporation.

Due to the facile, highly selective nature of copper-catalyzed cycloaddition of terminal alkynes and azides (106, 107) (*i.e.*, click chemistry), numerous groups have employed this reaction to label, enrich, and quantify glycopeptides. A benchmark study, isotope-targeted glycoproteomics (IsoTaG), demonstrated the ability to incorporate azide-containing sugars into nascent glycans (108, 109, 110). This azide sugar was then ligated to an acid-labile, isotopically labeled biotin tag with a terminal alkyl group for glycopeptide enrichment with streptavidin beads. The biotin tag was then cleaved, leaving behind the isotopic group, which could then be used for targeted mass spectrometry due to the characteristic mass shift against isotopic partners. The combined efficiencies of azide sugar incorporation and biotin-streptavidin enrichment presented a powerful strategy for quantitative glycomics and glycan/glycopeptide enrichment. Though this method has difficulty in complete characterization of N-glycans—due to the unpredictable composition of sialic-acid-containing glycopeptides—the authors successfully elucidated 32 N-glycopeptides with additional 156 partial assignments and completed characterization of more than 500 O-glycopeptides. The shortcomings in N-glycopeptide detection were addressed in a later study that incorporated alkyne-sugars rather than azido-sugars, which facilitated greater access to N- and O-glycopeptides alike with 156 and 578 confident identifications, respectively (111). A key benefit of employing IsoTaG is the accompanying software, IsoStamp (112), which aids in spectral deconvolution and quantitation. Such benefits are replicated in the study from Qin *et al.* (113) that detailed O-glycopeptide analysis through isotope-tagged cleavable linker (isoTCL) and quantitation using MaxQuant. Though quantitative accuracy was still achieved, manual confirmation of heavy/light pairs must be performed, bolstering the value of IsoTag and IsoStamp that eliminate the need for validation. Finally, in order to eliminate the harsh solution conditions associated with acid-labile chemical probes, a photocleavable biotin tag for O-GlcNAcylated glycopeptide quantification was developed by Li *et al.* (114). This study localized 419 and 276 O-GlcNAcylation sites from sorafenib-sensitive and sorafenib-resistant HepG2 cells, respectively, 262 of which were not previously reported.

### Isotopic Labeling

Following the trend seen in glycan analyses, isotopic labeling is a method of choice in glycopeptide quantitation due to the well-characterized nature of numerous peptide labeling strategies. As dimethyl labeling is a highly facile method for peptide derivatization and employs reagents that are not cost-preventative, numerous reports detail the utility of dimethyl labeling in lower-throughput relative glycopeptide quantitation experiments (115, 116). Novel applications include the association of altered glycopeptide glycosylation profiles with pancreatic cancer (117), glycoproteomic profiling in triple-negative breast carcinomas through analysis of deglycosylated peptides (118), quantitative comparisons of sialic-acid-containing glycopeptides in human embryotic and neural stem cells (119), and employing deglycosylated peptides to determine changes in site occupancy rates between normal liver and hepatocellular carcinoma (HCC) liver tissues (120). Further development of this strategy has been seen in the employment of diethyl labeling of glycopeptides (121, 122, 123), which reduces retention time differences and quantitation errors by replacing and incorporating heavy carbon in place of deuterium.

Though chemical labeling strategies such as dimethyl labeling are facile in nature, reagent purity and labeling efficiency are persistent factors that reduce the overall efficiency and accuracy of glycomic quantitation. However, in search of avenues for isotope incorporation with high efficiency and no need for intensive synthesis, researchers have capitalized on the mechanism of proteolytic cleavage to incorporate more advantageous stable isotopes, such as ^18^O. A novel strategy for ^18^O stable isotope labeling (TOSIL) of deglycosylated peptides was presented by Liu *et al.* (124) and later adapted for use in complex samples (125). By performing trypsin digestion in the presence of heavy water, the newly formed C terminus will be labeled with two ^18^O atoms. PNGase F treatment of the formed peptides will result in additional ^18^O atom being incorporated during the transition of the Asn modification site to Asp. This strategy was employed for accurate quantitation of glycosylation profiles between innovator and biosimilar antibodies (126). Though this method retains no glycan-specific information, the authors employed selective lectin enrichment prior to glycan release to generate glycopeptide subgroups to evaluate topical modification changes. Validated in comparisons of normal and HCC liver cells, this method demonstrated high quantitative accuracy across the dynamic range and complete isotopic envelope separation. To evaluate the utility of the original TOSIL method for N-glycoproteome quantitation, Liu *et al.* (127) employed TOSIL in tandem with lectin microarrays to reveal potential biomarkers in HCC metastasis.

In addition to these innovations, numerous groups have developed novel chemical tags useful for glycopeptide labeling, which are easily translated to quantitative experiments after synthesizing the deuterium isotopologue. For example, Kurogochi and Amano (128) employed benzoic acid N-succinimidyl ester to enhance ionization efficiency of glycopeptides in MALDI-based quantitative experiments, while Pabst *et al.* (129) later determined galactosylation and sialylation patterns in Immunoglobulin G (IgG) glycopeptides in both ESI and MALDI regimes through derivatization with succinic anhydride. As routine proteolytic digestion involves reduction of disulfide bonds and protection through alkylation, reports have detailed the utilization of these processes for direct peptide labeling. Kim *et al.* (130) conceived the use of isotope-coded carbamidomethylation to label deglycosylated peptide species in tandem with free thiol protection, while Zhao *et al.* (131) employed isotopic dithiothreitol to label O-glycosylation sites after beta-elimination to produce deglycosylated peptides. These are attractive methods for peptide quantification as it does not involve subsequent sample handling or cleanup beyond those used in routine digestion workflows. Validated strategies such as these provide a litany of facile labeling strategies for relative glycopeptide quantitation but are inherently limited by low channel number and the inability to facilitate absolute quantitation. In remedy, recent reports have demonstrated the utility of isotopic internal standard peptides for absolute quantitation and novel application.

First, Zhu *et al.* (132) reported a strategy to determine absolute quantitation of glycosite occupancy in experiments using deglycosylated peptide abundance compared with isotope-coded synthetic peptides. Noting deamidation of Asn residues is shown to occur spontaneously during sample preparation and therefore skew quantitative comparisons of deglycosylated peptides, this work synthesized isotopic deglycosylated peptide partners. This allowed site occupancy to be reliably quantified by subtracting the concentration of nonglycosylated protein from total protein concentration. Later, Roy *et al.* (133) reported a strategy for absolute quantitation of IgG subclasses by synthesizing isotopic glycopeptides using Asn-GlcNAc residues that display no difference in retention time from glycopeptides produced during digestion. This method could be readily expanded due to the flexibility of peptide synthesis and accuracy of internal standard calibration. Finally, Nilson *et al.* (134) reported a method to quantify the recently reported amyloid-β (Aβ) glycopeptide as well as unmodified Aβ in cerebrospinal fluid. Though Aβ glycopeptide contains a rare Tyrosine O-glycosylation (Tyr-GalNAc) and internal standards require intensive derivation prior to peptide synthesis, the report accurately quantifies differences in glycosylated Aβ-15 and Aβ-17 fragments across 20 Alzheimer’s disease patients and 20 nondemented controls. As synthetic peptide production evolves and the reliability and accuracy of multi- and parallel-reaction monitoring strategies continue to improve, these reports are likely to serve as a basis for broad absolute glycopeptide quantitation.

### Isobaric Labeling

The multiplexing capacity of isobaric peptide labeling provides a high degree of experimental accuracy and throughput in quantitative proteomic investigations. Traditional methods such as isobaric tags for relative and absolute quantitation (iTRAQ) and tandem mass tags (TMT) have been widely employed for glycoproteomic profiling in various biological samples. Among these, iTRAQ has been utilized for N-glycopeptide analyses in neurodegenerative diseases (135) and cardiac hypertrophy (136), analyzing glycopeptides to profile the glycoproteome of human tear fluid (137), and interrogating deglycosylated peptides to reveal dynamic glycoprotein regulation in maize seedlings (138), representing the utility of iTRAQ to further glycomic experimentation. TMT has seen even greater utility in quantitative experiments as they have been applied to evaluate glycopeptide perturbations in HCC patient plasma (139), pancreatic cancer serum (140), aggressive prostate cancer cell lines (141) and urinary profiles of prostate cancer patients (142), human cell surfaces (143), cerebrospinal fluid (144) (glycopeptides and deglycosylated peptides), and PNGase F-resistant N-glycopeptides (145), as well as the evaluation of glycopeptide enrichment strategies (146) *via* direct analysis through ETD (147) and strategies for simultaneous phosphopeptide and glycopeptide quantitation (148). iTRAQ and TMT are attractive strategies for those seeking reliable relative glycopeptide quantitation, with added benefits of well-documented workflow, commercial availability, and quality control. However, the steep cost of these commercial reagents makes them unsuitable for use during method development or exploratory studies and is not amenable to bespoke method alteration. Recently, a promising alternative was presented that allows for a significant reduction in cost, facile in-house synthesis, and a high degree of flexibility for method experimentation.

**Fig. 5:**
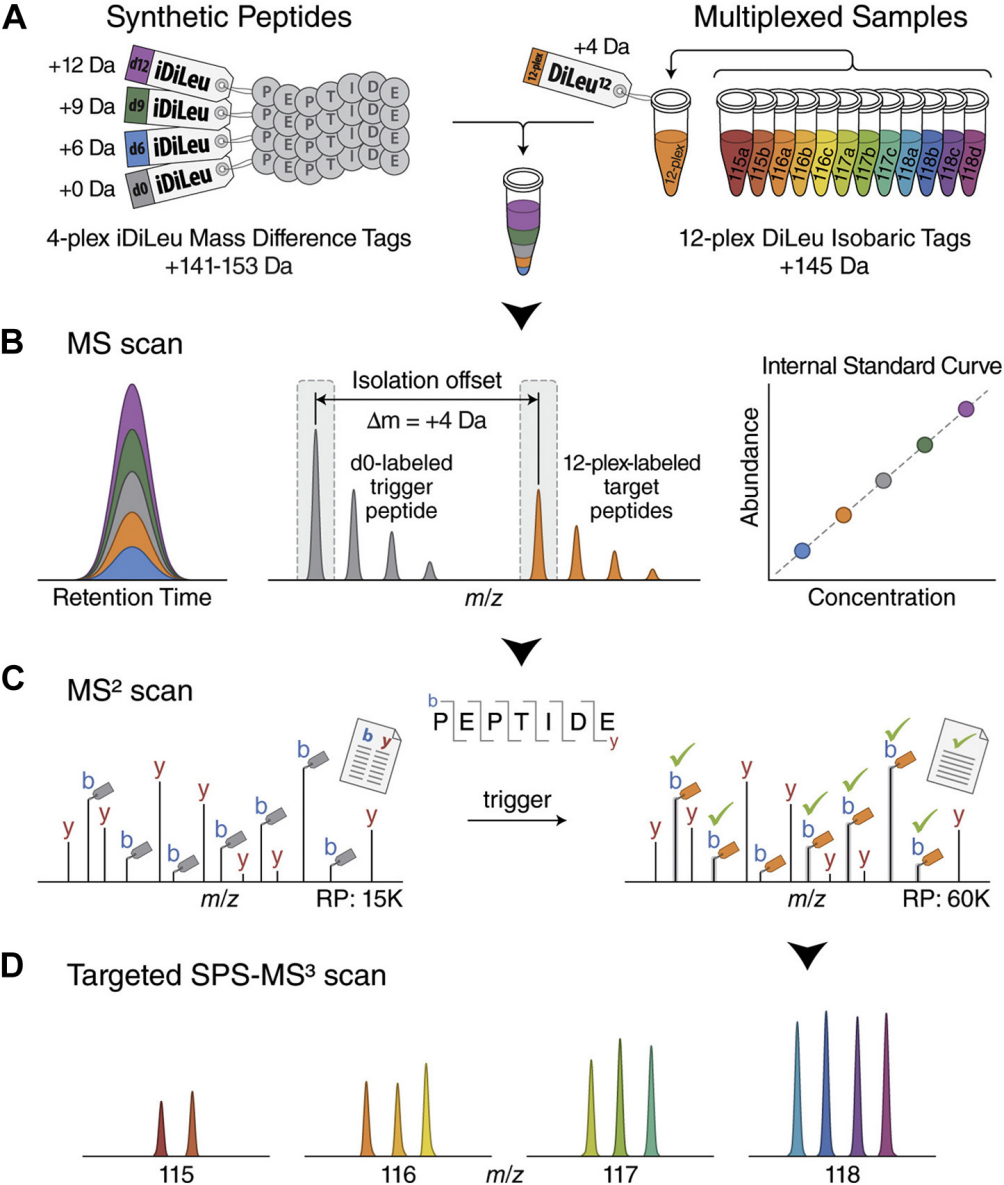
**Schematic illustration for the HOTMAQ method.***A*, synthetic peptides are labeled with four-plex iDiLeu at different concentrations and spiked into 12-plex DiLeu-labeled analytes. *B*, labeled peptides are detected with identical chromatographic elution profiles as five precursor ion clusters. The iDiLeu labeled-synthetic peptides are used to generate internal calibration curves to quantify the total amount of multiplexed target peptides. iDiLeu d0-labeled synthetic trigger peptides and multiplexed DiLeu-labeled target peptides are separated in MS^1^ spectra by a mass offset of 4.01 Da, which enables synthetic trigger peptides to initiate quantitative analysis of target peptides *via* MS^2^ regardless of target peptide precursor abundances. *C*, real-time MS^2^ analysis of d0-labeled synthetic peptides by matching MS2 spectrum to a product mass inclusion list unambiguously triggers fragmentation of 12-plex DiLeu-labeled target peptides in a predefined monitoring window. Acquisition parameters alternate between a low-resolution scan for monitoring d0-labeled trigger peptides and a high-resolution scan for quantifying 12-plex DiLeu-labeled target peptides. Fragment ions of 12-plex DiLeu-labeled target peptides are selected for synchronous precursor selection (SPS)-MS^3^ analysis. *D*, the relative abundance of each 12-plex DiLeu-labeled peptide is accurately determined by targeted SPS-MS3 acquisition at a resolving power of 60K (at *m/z* 200). The absolute amounts of target peptides are quantified by integrating the total amount obtained using the standard curve. Adapted from Zhong *et al.* (156) with permission.

N,N-dimethyl leucine (DiLeu) isobaric tags were originally presented in 2010 by Xiang *et al.* (149) as a novel four-plex strategy for quantitative proteomics. With commercial leucine as the starting material, each reporting channel is synthesized in no more than two simple reactions, each of which employs commonly available reagents—emphasizing cost-efficiency. Due to the comparable performance when evaluated against iTRAQ, DiLeu was expanded to a five-plex platform for absolute quantitation (iDiLeu) (150) and an eight-plex relative quantitation strategy that maintained the overall ease of synthesis from the original report. DiLeu was further developed to facilitate 12-plex relative quantitation (151), utilizing mass-defect principle and higher-resolution instrumentation that is becoming more readily available, and this strategy was then coupled with dimethyl labeling, producing an effective 24-channel strategy for relative quantitation (152). DiLeu isobaric labels have been evaluated in a number of proteomic and peptidomic experiments (150, 152, 153, 154, 155) and have also been developed into an absolute quantitation strategy. Hybrid offset-triggered multiplex absolute quantification (HOTMAQ) combines four-plex iDiLeu with 12-plex isobaric tags to create an internal calibration curve at the MS1 level in tandem with identification of peptides at the MS2 level (156) (Fig. 5). This strategy provides up to a 12-fold increase in throughput during absolute quantitation experiments.

Of interest, DiLeu tags were recently applied for site-specific characterization and quantitation of N-glycopeptides in PANC1 pancreatic cancer and PKM2 knockout breast cancer cells (157). As sialylated glycans are known to be upregulated in various cancers and show distinct expression across lifetime, this study provided an early report on the most efficient strategy for sialylated N-glycopeptide extraction and enrichment. Method validation in PANC1 experiments revealed 1067 N-glycopeptides, 311 glycosites, and 88 glycan compositions from 205 glycoproteins. Quantitative evaluations of PKM2 cells provided evidence that N-glycosylation signaling pathways are tightly regulated by cellular metabolism, with 484 N-glycopeptides quantified and 81 showing significant changes in expression. As this method offers comparable performance to the hallmark commercial methods of TMT and iTRAQ, as well as providing an avenue for mass-defect-based proteomics (65), development and employment of DiLeu isobaric labels are a beneficial strategy for accurate, cost-effective proteomic and glycoproteomic quantitation with great room for further implementation.

### Label-Free and MS Reaction Monitoring

While a small number of reports detail the implementation of mathematical modeling to facilitate accurate, label-free quantitation of glycopeptides—such as that detailed by Mayampurath *et al.* (158)—glycopeptide quantitation has benefited greatly from the implementation of PRM and MRM. Similar to strategies implemented for glycan analyses, reaction monitoring of glycopeptides does offer high quantitative accuracy and improved sensitivity, but requires deeper consideration. MRM analysis requires effective ionization of glycopeptides and the production of reproducible, quantifiable fragments. As hydrophilic glycans reduce the overall ionization efficiency and the heterogeneity of glycosylation divides the intensity of glycopeptides across several glycoforms (86), enrichment strategies are often required to improve detectability against complex peptide backgrounds and avoid loss of minor glycoforms within the mixture (159). However, these strategies have not prevented the successful implementation of MRM for numerous novel investigations. Of note, MRM has enabled successful quantification of differential expression of IgG subclass glycosylation (160), haptoglobin glycoforms (161, 162), and core fucosylation (163) in liver disease, profile changes in galactosylation and sialylation in rheumatoid arthritis (RA) patients (164) quantify glycoproteins in esophagus disease (165), reveal alterations in murine immunoglobulin glycoforms (166), characterizing the function and importance of UDP-GlcNAc transporter (167), and quantitation of Golgi-resident glycosylation enzymes from cultured human cells (168). In addition, researchers have also detailed methods for glycopeptide quantitation in a range of human biofluids such as human serum (169, 170) and liver cancer plasma (171). Pinpointing some standouts, Srikanth *et al.* provided a quantitative method that combined ^18^O labeling and MRM, Jian *et al.* (172) established the feasibility of top-down glycoprotein characterization when protein length is short, Hammura *et al.* (173) detailed a method to both synthesize and quantify rare bisecting N-glycans in therapeutic antibodies, and van der Burgt *et al.* (174) implemented a strategy to quantify sialic acid linkage isomers of prostate-specific antigen (PSA). The later study also provides a topical comparison of various analytical methods for linkage isomer analysis on the basis of throughput, robustness, quantification ability, recognition of glycoforms, and isomer separation, which may be of interest to some readers.

In addition to these above reports, Lebrilla and coworkers have developed strategies to expand the use of MRM for glycopeptide analysis. Offering numerous reports of MRM analysis that identify and quantify immunoglobulin classes (*i.e.*, IgG, IgA, IgM) and their glycosylation profiles (175, 176), as well as quantify site-specific glycosylation in recombinant antibody drugs (177), this group has also provided accurate quantitation of human milk protein glycoforms (178) and evaluated the differential expression of serum glycoproteins to serve as biomarkers in ovarian cancer (179). Furthermore, improvements in implementing dynamic multiple reaction monitoring (dMRM) have been reported. Although conventional MRM analyses are highly specific, minimizing the ailments surrounding coeluting peptides that may cause ion suppression and fail to identify low-abundance analytes, monitoring specific targets and transitions over the entire chromatographic timeframe severely reduces the number of analytes that may be quantified. As such, Li *et al.* (180) hypothesized that retention time may be leveraged to reduce the time spent searching for selected precursor and transition masses, thereby increasing the number of novel species quantified. Employing multienzyme standard protein digestion to produce smaller glycopeptides and increase sample coverage, this strategy first employed orbitrap-based analysis of enriched glycopeptides that were identified by Byonic (vide infra). In addition to the identified glycopeptides, the authors imputed missing values for undetected species by generating *in silico* transition masses and predicting retention time according to the relative hydrophobicity of the glycopeptides. Using the retention times, precursor masses, and unique transitions of all identified and suspected analytes to build a dMRM method, the authors were able to quantify nearly 700 glycopeptides in a single 50-min LC run, which was then validated on human serum samples. With low femtomolar limits of detection and quantification, this method illustrates the utility of MRM for complex sample quantification and the ability to accommodate higher throughput. Taken together, the specificity, enhanced sensitivity, and uncompromised quantification accuracy of MRM are an attractive strategy for glycopeptide and glycoprotein quantitation with much room left for novel innovation and application.

**Fig. 6:**
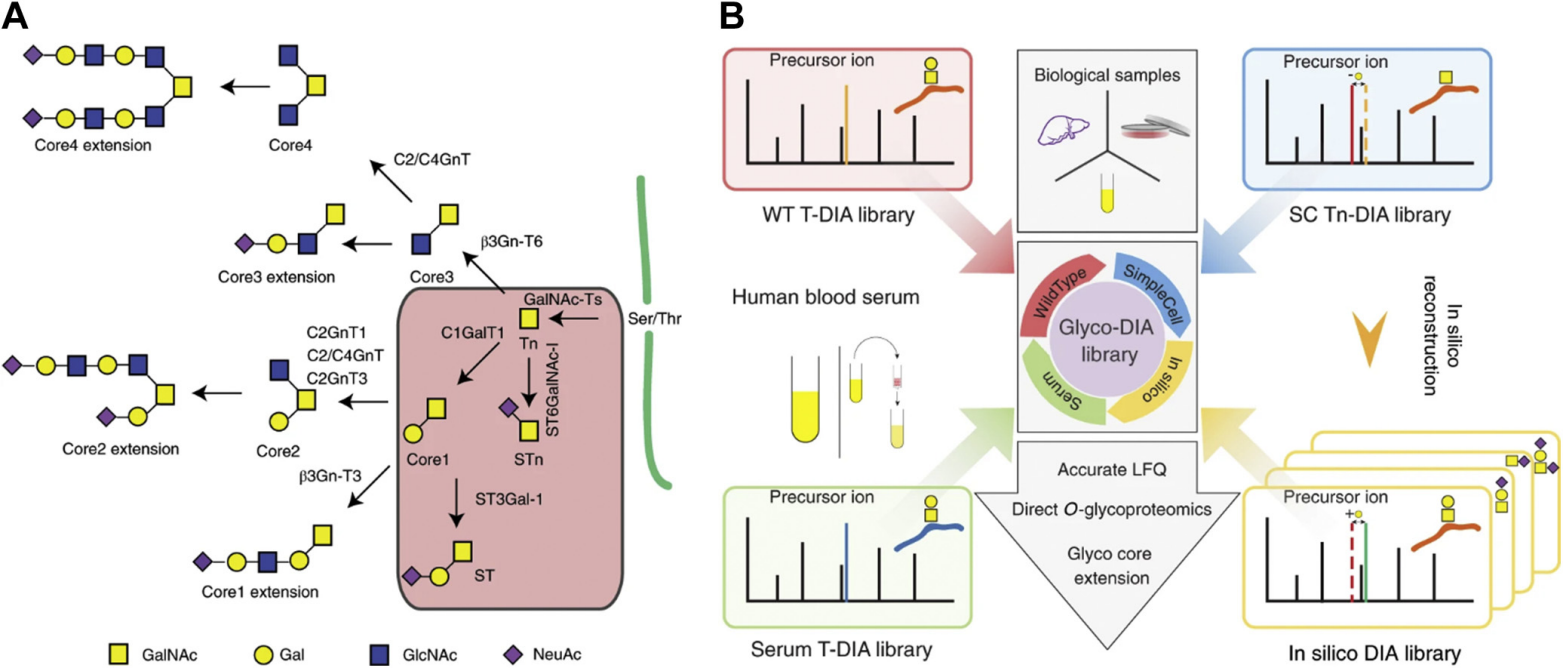
***A*, the major biosynthetic steps and enzymes involved in core1 to 4 *O*-glycan structures with extensions and capping by sialic acid are illustrated.***B*, overview of Glyco-DIA libraries. The Glyco-DIA library consists of several sublibraries, including Tn-DIA libraries from SC cell lines, T-DIA libraries from WT cell lines, T-DIA library from human blood serum, and *in silico* expanded libraries. LFQ, label-free quantification. Reprinted from Ye *et al.* (183) with permission from the author.

As typical limitations in glycopeptide detection and identification include low concentration of glycopeptides within proteolytic mixtures and poor ionization efficiency, many glycopeptide species are overlooked and not selected for MS/MS fragmentation in DDA experiments. For this reason, DIA has gained steady traction in broad proteomic and glycoproteomic experiments for its ability to expand profiling depth and select low-lying precursor masses, offering potential remedy to the low-throughput of MRM analyses (181). Typical DIA experiments such as SWATH-MS (*i.e.*, sequential window acquisition of all theoretical fragment ion spectra mass spectrometry) require user definition of *m/z* windows to be used for fragmentation. As most peptides are found within 400 to 1250 *m/z*, common practice is to set consistent window sizes (∼25–36 *m/z*) over this range. However, due to the large mass addition of glycans, glycopeptides are not evenly distributed along this range and are concentrated between 950 and 1200 *m/z*. As such, Zhou and Schulz (182) validated a more effective strategy, GP-SWATH that narrows selection window width across the glycopeptide region to provide more accurate and robust glycopeptide detection and quantification. A notable limitation in DIA analysis is the deconvolution of tandem MS spectra as DIA experiments commonly lose precursor information, making identification of posttranslationally modified peptides a challenge—especially for O-glycopeptides. Offering alleviation of this ailment, Ye *et al.* (183) recently established Glyco-DIA, a strategy to provide enhanced O-glycopeptide identification and quantitation. As illustrated in Figure 6, this method constructs spectral libraries from numerous DDA experiments, which can be expanded *in silico* to provide missing values. Evaluation of this methodology revealed significantly improved performance of O-glycopeptides in direct analyses with even greater benefit in runs performed after enrichment. Though the authors state limitations in this method such as biasing toward abundant O-glycopeptides in DDA experiments, Glyco-DIA may be rapidly expanded for O-glycoproteome coverage and tailored for individual, targeted analyses.

### Software Advances

Accurate glycopeptide annotation is dependent on efficient glycan and peptide fragmentation, as the high compositional complexity of all glycans and the challenges in glycosite assignment of O-glycans can easily be misinterpreted and result in false identifications. Though few studies have evaluated the efficacy of decoy glycopeptide databases (184, 185, 186, 187), numerous advances have been made in developing open-source and commercial software capable of adept peptide annotation and quantitative comparisons. Premier Biosoft International provided early access into spectral deconvolution for glycan analysis. Touting a robust relational database of glycans and glycoproteins, support for MALDI and ESI file formats, glycopeptide qualitative analysis, built-in functionality to process TMT-based quantitative information, and the ability to assign glycan structure from MS^n^ data, SimGlycan remains a relevant and effective tool for glycomic investigation. Bern *et al.* (188) (Protein Metrics) introduced Byonic in 2012 for peptide and protein identification, which remains a premier method for glycopeptide identification. Following suit, Protein Metrics later introduced Byologic to facilitate an identification/quantitative analysis pipeline, which has been validated in a number of glycopeptide studies (189, 190). As these licensed commercial software packages may be cost-preventative and not widely employed by individual users, open-source alternatives have been reported. LaCyTools (191) and GlycopeptideGraphMS (192) are python-based utilities that have reported improved glycopeptide identification and quantitation, while GPSeeker (116) facilitates structural N-glycoproteomics by integrating previously reported software from the same research group (193, 194, 195). SugarQb (145, 196) was developed to provide glycan and glycopeptide insights within the Proteome Discoverer (Thermo) environment. An alternative to working within Proteome Discoverer is presented by Maxwell *et al.* (197) in their development of GlycReSoft. Building on their validated strategy for targeted glycan analyses, Manatee (198), GlycReSoft implements a data deconvolution algorithm to enable the rapid extraction and confidence scoring of glycan and glycopeptide identifications in both supervised and unsupervised analyses. In addition, GlycReSoft provides a user-friendly web-based application that can also leverage distributed computation to accommodate broad search space. The same research group later validated novel tools for increased glycomic profiling (199, 200), which utilized knowledge of biosynthetic pathways to improve glycan feature recognition. Finally, Integrated GlycoProteome Analyzer (I-GPA) enables global characterization of site-specific structural features and reliable, automated label-free quantitation (201).

One freely-accessible alternative that has gained much attention is pGlyco (202) and its latest iteration, pGlyco 2.0 (203). As the initial software was a useful tool for glycopeptide spectra deconvolution, the authors state the need for expansion due to the existing limitations in high-throughput tools for peptide and glycan identification, the inability of current software to provide built-in manual interpretation and validation, and most notably, the lack of robust quality control and FDR estimation that drastically underperform in adjacent bioinformatic tools. The latter point is echoed by Park *et al.* (201), who provided topical comparisons of FDR approximations through GlycoFraqWork (204), GP Finder (205), Sweet-Heart (206), and GPS (207). Further, as stepped collisional energy (SCE) dissociation was nascent at the time of publication but was shown to outperform single regime (*i.e.*, CID, HCD, and ETD) and hybrid fragmentation modes (*i.e.*, ETciD and EThcD), pGlyco 2.0 provided early access to using SCE for broad glycopeptide analysis. pGlyco 2.0 validated an improved FDR estimation through isotope-based and entrapment-based strategies. Complete details of these strategies may be read within (203), but performing database searches of the same data (*i.e.*, yeast cell lysate digest) using pGlyco 2.0 provided <1% FDR while Byonic resulted in >19%, and every identification may be visually inspected in pGlyco 2.0 using the built-in gLabel software. In terms of raw performance, five mouse tissues (brain, heart, kidney, liver, and lung) were analyzed and subjected to pGlyco searching, which revealed 10,009 site-specific glycans on 1988 glycosites from 955 glycoproteins with quantitation enabled through pQuant. pGlyco was then used to re-evaluate the previously discussed NGAG dataset (61) that used GPQuest as the search engine and revealed a 97% increase in glycopeptide identifications from the same data. Though pGlyco 2.0 was not heavily utilized for O-glycopeptide discovery, topical analyses of asialofetuin standard glycoprotein revealed reliable N- and O-glycopeptide identifications, indicating analytical potential. Taken together, pGlyco 2.0 presents a powerful, open-source option for robust glycopeptide identification.

## Conclusions and Future Directions

The field of glycan and glycopeptide quantitation has experienced tremendous growth over the past decade. Widely accepted as an area of significant analytical challenge, the numerous creative strategies demonstrated above have proven successful as they directly address areas of topical concern in glycomic analyses. Ionization efficiency may be improved through glycan permethylation or by employing labels that increase hydrophobicity or impart permanent positive charge. The need for effective enrichment was addressed by methods that incorporate azide-containing sugars during cell culture for use in click chemistry labeling experiments. And instrumental functionality such as multiple reaction monitoring and DIA alleviate consequences of low glycopeptide abundance within a proteolytic sample. However, though these examples present significant advances in glycomic analysis, many improvements are still needed.

As pursuit of quantitative glycomics increases, researchers will be left searching for higher-throughput methodologies and inevitably seek strategies for absolute quantitation. Methods presented above will lay the foundation for these new techniques, most likely seeing numerous strategies used in tandem, such as the workflow demonstrated in HOTMAQ (156). Additionally, coverage of the glycome and glycoproteome will benefit from improvements in sensitivity. Lower- and nanoflow, chip-based technologies facilitate much greater signal response from glycan and glycopeptide species and are likely to be invaluable strategies moving forward. As well, capillary electrophoresis is likely to see greater implementation in glycomics investigations, owing to the extremely low sample consumption, ability to resolve isomeric mixtures and ultrahigh resolution. Alternatively, researchers may choose to boost glycan and glycopeptide abundance at the MS^1^ level by using methods more amenable to the labeling strategy employed, such as that shown in BASIL (boosting to amplify signal with isobaric labeling) (208). Finally, computational tools and software capable of accurately deconvoluting and correctly assigning glycomic observations will be an area of continual need. Decoy database creation and implementation will see greater utilization as quantitative glycomics gains popularity, and resource bottlenecks (*e.g.*, CPU processing speed and available cores) must be alleviated as access to the glycome increases.

Taken together, the field of quantitative glycomics is a space rich in invention, novel implementation, and discovery. Numerous labeling strategies have enabled facile, accurate investigations of disease-relevant glycoproteins and are well suited to uncover future biomarkers and discern symptomatic protein profiles. The developments in instrumental capability over the next several years are likely to provide greater expansion in chemical labeling experiments and possibly enable greater implementation of label-free quantitative strategies. But no matter the direction, quantitative glycomics and glycoproteomics will remain an area of significant active focus for years to come, with numerous challenges still to be presented and overcome.

## Conflict of interest

Authors declare no competing interests.
